# Medication safety issues in the emergency department

**DOI:** 10.1186/1757-7241-20-S2-P31

**Published:** 2012-04-16

**Authors:** Suheil Andreas Salamon, Charlotte Arp Sørensen, Tina Birkeskov Axelsen

**Affiliations:** 1 Regionshospitalet Randers, Skovlyvej 1, 8900 Randers, Denmark

## Background

The effect of clinical pharmacist conducted medication history, registration in electronic module and reconciliation, was studied in unscheduled patients admitted to our emergency department.

## Methods

A prospective study which enrolled unscheduled patients presenting to a regional emergency department. In the control group, medication history, registration and reconciliation was conducted by junior doctors. Duration for medical journal record and medication registration was noticed separately. In the intervention group, clinical pharmacists conducted medication history, registration and reconciliation. Junior doctors conducted only medical journal record. Time was noticed for both groups.

## Results

135 consecutive patients were enrolled: 44 patients in the control group and 91 patients in the intervention group. In the intervention group, there was a significant reduction of at least 15 minutes (p = 0.005548), when junior doctors only conducted medical journal record, without medication history, registration and reconciliation. Clinical pharmacists used 38.5 minutes (CI 95 %: 34.5 – 42.5), for conduction of medication history, registration and reconciliation.

## Conclusion

32 unscheduled patients are admitted to our emergency department daily. We conclude that, an average of approximately 8 hours will be added to junior doctor’s real time patient contact and treatment. The quality of medication history, registration and reconciliation, is also improved, when conducted by a clinical pharmacist.

